# Serum immunoglobulin G, M and A response to *Cryptosporidium parvum *in *Cryptosporidium*-HIV co-infected patients

**DOI:** 10.1186/1471-2334-9-179

**Published:** 2009-11-18

**Authors:** Kirti Kaushik, Sumeeta Khurana, Ajay Wanchu, Nancy Malla

**Affiliations:** 1 Department of Parasitology, Postgraduate Institute of Medical Education and Research, Chandigarh-160 012, India; 2 Department of Internal Medicine, Postgraduate Institute of Medical Education and Research, Chandigarh-160 012, India

## Abstract

**Background:**

*Cryptosporidium parvum*, the protozoan parasite, causes a significant enteric disease in immunocompromised hosts such as HIV patients. The present study was aimed to compare serum IgG, IgM and IgA responses to crude soluble antigen of *C. parvum *in HIV seropositive and seronegative patients co-infected with *Cryptosporidium *and to correlate the responses with symptomatology.

**Methods:**

*Cryptosporidium parvum *specific serum antibody (IgG, IgM and IgA) responses were assessed by ELISA in 11 HIV seropositive *Cryptosporidium *positive (Group I), 20 HIV seropositive *Cryptosporidium *negative (Group II), 10 HIV seronegative *Cryptosporidium *positive (Group III), 20 HIV seronegative *Cryptosporidium *negative healthy individuals (Group IV) and 25 patients with other parasitic diseases (Group V).

**Results:**

A positive IgG and IgA antibody response was observed in significantly higher number of *Cryptosporidium *infected individuals (Gp I and III) compared to *Cryptosporidium *un-infected individuals (Gp II, IV and V) irrespective of HIV/immune status. Sensitivity of IgG ELISA in our study was found to be higher as compared to IgM and IgA ELISA. The number of patients with positive IgG, IgM and IgA response was not significantly different in HIV seropositive *Cryptosporidium *positive patients with diarrhoea when compared to patients without diarrhoea and in patients with CD4 counts <200 when compared to patients with CD4 counts >200 cells/μl.

**Conclusion:**

The study showed specific serum IgG and IgA production in patients infected with *Cryptosporidium*, both HIV seropositive and seronegative as compared to uninfected subjects suggesting induction of *Cryptosporidium *specific humoral immune response in infected subjects. However, there was no difference in number of patients with positive response in HIV seropositive or seronegative groups indicating that HIV status may not be playing significant role in modulation of *Cryptosporidium *specific antibody responses. The number of patients with positive IgG, IgM and IgA response was not significantly different in patients with or without history of diarrhoea thereby indicating that *Cryptosporidium *specific antibody responses may not be necessarily associated with protection from symptomatology.

## Background

*Cryptosporidium parvum*, the protozoan parasite, causes a significant enteric disease in immunocompromised hosts such as HIV patients. Severe chronic infections may develop in immunocompromised hosts with lymphocyte or gammaglobulin deficiencies which suggest that both cell mediated and humoral immune responses are involved in resolution of infections and development of protection [1]. Serologic surveys in immunocompetent individuals or HIV/AIDS patients show varying levels of anti *Cryptosporidium *antibodies. Detectable IgG levels against *Cryptosporidium *were reported in 86% in Australia [2], 26% in Britain [3], 64% in Peru [4] and 64% in Venezuela [4] in healthy individuals. A previous study [5] reported *Cryptosporidium *specific positive IgG response in 5 (100%) HIV patients and 12 (100%) immunocompetent patients. In another study [6] IgG antibodies were observed in 13 out of 15 (86.7%) immunocompetent and all the 26 (100%) AIDS patients studied and IgM response was observed in 14 out of 15 (93.3%) immunocompetent and 4 out of 26 (15.4%) HIV positive patients. A positive IgG and IgM response was reported in all 16 (100%) immunocompetent and 24 (100%) AIDS patients studied [7]. In a similar study [8] positive IgG, IgM and IgA response was observed in all the 4 (100%) immunocompetent and 4 (100%) HIV positive patients, studied. The role of antibody responses in protection is still not clear. In the study conducted in Alabama [5], antibodies to *Cryptosporidium *were detected in sera from 5 AIDS subjects with persistent cryptosporidiosis. The fact that some AIDS patients with persistent symptomatic infection with *Cryptosporidium *have a high antibody titre to *C. parvum *supports the theory that specific serum antibody alone is not sufficient to control the infection [9]. Studies comparing the IgG, IgM and IgA response in HIV seropositive and seronegative patients to *C. parvum *are scarce and reported in very limited numbers of subjects. In India the number of HIV infected individuals is growing at an alarming rate with 2.47 million people infected with HIV till the end of year 2006 [10]. Although, *Cryptosporidium *has been reported in 4.6%-12% HIV patients from different geographical areas in India [11-17], reports regarding IgG, IgM or IgA response to *C. parvum *in HIV seropositive or seronegative subjects are totally lacking. The present study was aimed to compare serum IgG, IgM and IgA responses to crude soluble antigen of *C. parvum *in HIV seropositive and seronegative patients co-infected with *Cryptosporidium *and to correlate the responses with symptomatology.

## Methods

### Subjects

Two hundred and six HIV seropositive, 153 HIV seronegative and 50 normal healthy individuals without any history suggestive of cryptosporidiosis were enrolled in a previous study for detection of *Cryptosporidium *by stool examination with Ziehl-Neelsen [18], safranine methylene blue staining [19], antigen detection ELISA (RIDASCREEN Cryptosporidium, R-Biofarm, Germany) and a nested PCR targeting the small subunit rRNA gene specific for *Cryptosporidium parvum *[20]. Based on the results of this study, out of the subjects detailed above, 11 HIV seropositive *Cryptosporidium *positive (Group I), 20 HIV seropositive *Cryptosporidium *negative (Group II), 10 HIV seronegative *Cryptosporidium *positive (Group III) and 20 HIV seronegative *Cryptosporidium *negative healthy individuals (Group IV) were selected for the present study, from the Immunodeficiency clinic, the inpatient and outpatient departments of Nehru Hospital attached to Post Graduate Institute of Medical Education and Research, Chandigarh, a tertiary care hospital in North India. For determining the specificity of ELISA, 25 patients with other parasitic infections (4 each with toxoplasmosis and amoebiasis, 2 with ascariasis and 5 each with malaria, hydatid and neurocysticercosis, respectively (Group V) were also included in the study. Diagnosis of HIV was established as per National AIDS Control Organization (NACO) guidelines (WHO criteria adopted by NACO) [10]. After obtaining informed consent from each individual, the demographic characters such as sex, age, history of diarrhoea and any other relevant symptoms were recorded on standard proforma. HIV patients receiving anti-retroviral therapy were excluded from the study. About 3-5 ml venous blood was collected from each patient in vials without any anti-coagulant within 15 days of onset of infection. The person performing the serologic assays was not blinded to the clinical status of the patients.

### Antigens

*Cryptosporidium parvum *oocysts (Iowa strain) were obtained from NIH AIDS Research and Reagent Program. *Cryptosporidium parvum *crude soluble antigen was prepared by sonication of oocysts with few modifications [21]. Briefly, oocysts were washed thrice with PBS (15,000 rpm, 15 min), suspended in PBS, freeze-thawed 20 times, sonicated (twelve cycles of 30 seconds) and centrifuged (15,000 rpm, 15 min, 4°C). The supernatant was used as crude soluble antigen of *Cryptosporidium *(CCA) after estimating protein by Lowry's method.

### Enzyme-linked Immunosorbent Assay (ELISA)

Serum IgG, IgM and IgA responses to *Cryptosporidium *crude soluble antigen (CCA) were detected by Indirect ELISA closely based on standard techniques [22]. The optimum dilutions of the antigen, serum and anti human horse radish peroxidase (HRP) conjugate (Sigma-Aldrich, USA) were determined by checkerboard titration with known positive (pooled sera from 5 patients found positive for *Cryptosporidium *by staining techniques and confirmed with antigen detection and PCR) and 5 negative control sera (apparently healthy individuals, excluded for HIV seropositivity, *Cryptosporidium *and intestinal parasitic infections by stool examination).

Each well of the 96-well microtitre plate (Nunc Inter Med, Denmark) was coated with 100 μl optimum dilution of the CCA in carbonate bicarbonate buffer. Plates were incubated at 4°C overnight followed by washing thrice with PBS containing 0.02% Tween-20 (PBST). The non specific sites were blocked with 2%BSA in PBST and plate was incubated at 37°C for 1 hr followed by washing thrice with PBST. The doubling dilutions of the test, positive and negative control sera (1:10-1:40) were prepared in 1%BSA in PBST and 100 μl was added to each well. The plates were incubated at 37°C for 1 hr and again washed thrice with PBST followed by addition of 100 μl/well of optimum dilutions of the anti-human IgG, IgM or IgA conjugated with horse radish peroxidase in 1%BSA in PBST and incubated at 37°C for 1 hr. After the incubation, the plates were washed thrice with PBST and Ortho Phenylene Diamine (OPD) and H_2_O_2 _were added as substrate (100 μl/well). The plates were incubated in dark for 30 min and then the reaction was stopped by adding 3 M H_2_SO_4_. The absorbance of the contents of each well was read at 492 nm in an A4 ELISA reader (Eurogenetics, Tessenderle, Belgium).

The cut off absorbance value (Optical Density) for each dilution was determined by the mean absorbance of the 5 negative control sera plus 2 Standard Deviation (S.D). The test sera giving absorbances that were equal to and above the cut off O.D were considered ELISA positive at that dilution under the test. Each sample was tested in duplicate and mean O.D value of two tests was taken as reading of that sample.

At the threshold titres, the sensitivity of the ELISA was calculated [23] based on the assumption that all *Cryptosporidium *positive cases from group I and III were true cases.

### Ethical clearance

Ethical approval for the study was granted by the Ethical Committee of the PGIMER, Chandigarh, India.

### Statistical analysis

Difference between percentage positivity for antibody response in the groups was determined by Fisher's exact test. A p-value < 0.05 was taken as indicative of a statistically significant difference.

## Results

### Demographics

Demographic characters of the individuals for the study are detailed out in Table 1. Out of 11 HIV seropositive *Cryptosporidium *positive (Group I), 5 (45.5%) and out of 20 HIV seropositive *Cryptosporidium *negative (Group II) patients, 9 (45%) had diarrhoea. All 10 HIV seronegative *Cryptosporidium *positive (Group III) patients had diarrhoea. Out of these, 4 (40%) had undergone kidney transplantation (Group IIIa) and rest 6 were presumably immunocompetent (Group IIIb) (Table 1).

**Table 1:** Demographic profile of the individuals included for the study of anti-*Cryptosporidium *antibody responses

	Group	N	Age^a^, years	Males	Females	H/o diarrhoea	^b^Post Tx	CD4 count^a^, cells/μl
**I**	**HIV+Crypto+**	11	34.1 ± 8.4 (25-46)	7 (63.6%)	4 (36.4%)	5 (45.5%)	Nil	182.5 ± 119.3 (46-379)
**II**	**HIV+Crypto-**	20	34.2 ± 10 (25-64)	15 (75%)	5 (25%)	9 (45%)	Nil	198.6 ± 143 (30-583)
**III**	**HIV-Crypto+**	10	26.2 ± 16.5 (3.5-46)	6 (60%)	4 (40%)	10 (100%)	4 (40%)	Not available
**IV**	**HIV-Crypto-(healthy)**	20	26.9 ± 3.2 (23-35)	10 (50%)	10 (50%)	Nil	Nil	Not available
	**Total**	61	30.5 ± 10.1 (3.5-64)	38 (62.3%)	23 (37.7%)	24 (39.3%)	4 (40%)	-

^a^Mean ± SD (range)

^b^Post renal transplantation

On retrospective analysis, CD4 counts were available for all patients in Group I and 17 out of 20 patients in Group II. Median CD4 cell count was 182.5 cells/μl and 198.6 cells/μl for HIV seropositive *Cryptosporidium *positive and HIV seropositive *Cryptosporidium *negative patients, respectively (Table 1).

### ELISA optimization

The optimum concentration of the *C. parvum *crude soluble antigen (CCA) per well was 1 μg for IgG and IgA detection and 0.5 μg for IgM detection. The optimum conjugate dilutions were 1:30,000 for IgG detection and 1:1,000 for IgM and IgA detection. The optimum serum dilution was found to be 1:20. Results at this dilution are used for further discussion and analysis.

### IgG antibody response

IgG antibody response was found positive in all 11 (100%) HIV seropositive *Cryptosporidium *positive (Gp I) and 10 (100%) HIV seronegative *Cryptosporidium *positive (Gp III) including all 4 post-transplant (Gp IIIa) patients. Among *Cryptosporidium *negative subjects (Gp II, IV and V) only three (12%) patients with other parasitic infections showed positive response (Table 2).

**Table 2:** Number of patients (%) showing positive antibody response to *C. parvum *crude soluble antigen (CCA)

**Gp**.		Number studied	Number positive		
			IgG	IgM	IgA
**I**	**HIV+Crypto+**	**11**	11 (100)	2 (18.2)	7 (63.6)
**II**	**HIV+Crypto-**	**20**	Nil	Nil	4 (20)
**III**	**HIV-Crypto+**	**10**	10 (100)	2 (20)	5 (50)
**IIIa**	**HIV-Crypto+**	**4**	4 (100)	1 (25)	1 (25)
**IIIb**	**HIV-Crypto+**	**6**	6 (100)	1 (16.7)	4 (66.7)
**IV**	**HIV-Crypto-**	**20**	Nil	Nil	2 (10)
**V**	**Other parasitic diseases**	**25**	3 (12)	2 (8)	2 (8)
					
	**Sensitivity (%)**		100	19	57.1
	**Specificity (%)**		95.4	97	87.7
					
	**I vs II**		<0.0001	NS	<0.05
	**I vs IV**		<0.0001	NS	<0.01
**P**	**I vs V**		<0.0001	NS	<0.01
	**II vs III**		<0.0001	NS	<0.05
	**III vs IV**		<0.0001	NS	<0.05
	**III vs V**		<0.0001	NS	<0.05

### IgM antibody response

IgM response was found positive in 2(18.2%) patients in HIV seropositive *Cryptosporidium *positive (Gp I) and in 2 (20%) patients in HIV seronegative *Cryptosporidium *positive patients (Gp III). In the individuals not infected with *Cryptosporidium*, only 2 (8%) patients infected with other parasitic diseases showed antibody response (Table 2).

### IgA antibody response

IgA response was found positive in 7(63.6%) patients in HIV seropositive *Cryptosporidium *positive (Gp I) and in 5 (50%) in HIV seronegative *Cryptosporidium *positive patients (Gp III). In HIV seropositive *Cryptosporidium *negative (Gp II), 4 (20%) and in HIV seronegative *Cryptosporidium *negative healthy (Gp IV), 2(10%) subjects showed positivity while 2(8%) patients with other parasitic diseases (Gp V) were found positive (Table 2).

**Figure 1:**
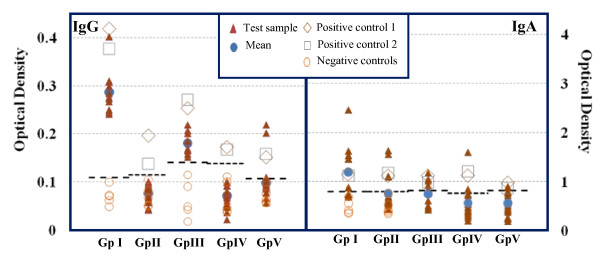
**IgG (left panel) and IgA (right panel) response to *Cryptosporidium parvum *crude soluble antigen as seen by ELISA**. Test sera were collected from HIV seropositive *Cryptosporidium *positive (Gp I), HIV seropositive *Cryptosporidium *negative (Gp II), HIV seronegative *Cryptosporidium *positive (Gp III), HIV seronegative *Cryptosporidium *negative apparently healthy (Gp IV) and HIV seronegative *Cryptosporidium *negative subjects infected with other parasites (Gp V). Dashed lines represent the cut-off OD for positivity.

A positive IgG and IgA antibody response was observed in significantly higher number of *Cryptosporidium *infected individuals (Gp I&III) compared to *Cryptosporidium *un-infected individuals (Gp II, IV & V) (p values < 0.0001 for IgG and <0.05 for IgA) irrespective of HIV status. There was no difference in number of patients found positive for IgG and IgA response between the two sub-groups in HIV seronegative *Cryptosporidium *positive patients i.e. between post-transplant (Gp IIIa) and presumably immunocompetent (Gp IIIb) patients (p > 0.05). No significant difference was observed in number of subjects showing positive IgM response in any of the groups (Table 2). IgG and IgA ELISA OD values for test samples, positive and negative controls, cut-off for positivity and mean OD for each group are shown in Fig 1.

### Sensitivity and specificity

Sensitivity and specificity of IgG, IgM and IgA ELISA by using *Cryptosporidium parvum *crude soluble antigen (CCA) was 100% and 95.4%, 19% and 97% and 57.1% and 87.7%, respectively (Table 2)

### Antibody responses Vs history of diarrhoea

Among the HIV seropositive *Cryptosporidium *positive patients, 11(100%), 2(18.2%) and 7(63.6%) were found positive for IgG, IgM and IgA antibodies respectively and out of these 5(45.5%), 0% and 4(57.1%) had a history of diarrhoea. Number of IgG, IgM or IgA positive patients with history of diarrhoea was not significantly different from that of patients without history of diarrhoea (p > 0.05).

### Antibody responses Vs CD4 counts

Among the HIV positive *Cryptosporidium *positive patients, 7(63.6%) out of 11 IgG positive patients had a CD4 count of < 200 cells/ul whereas 1 out of 2 (50%) IgM positive and 4 out of 7(57.1%) IgA positive patients had CD4 counts <200 cells/ul. Number of IgG, IgM or IgA positive patients with CD4 counts <200 cells/μl was not significantly different from that of patients with CD4 counts >200 cells/μl (p > 0.05).

## Discussion

In the present study, *Cryptosporidium *specific serum antibody (IgG, IgM and IgA) response was studied in doubling serum dilutions (1:10-1:40) to assess and compare the response in different study groups. We found that 1:20 dilution had the maximum sensitivity and specificity and was subsequently taken for interpretation of results.

In the present study, IgG antibody response was found positive in all 11 (100%) HIV seropositive *Cryptosporidium *positive (Gp I) and 10 (100%) HIV seronegative *Cryptosporidium *positive (Gp III) including 4 (100%) post-transplant (Gp IIIa) patients while in the *Cryptosporidium *negative groups (Gp II, IV and V), 3 (12%) patients with other parasitic infections showed positive response. In the present study, no difference was observed between number of patients showing positive response in HIV seropositive *Cryptosporidium *positive (Gp I) and HIV seronegative *Cryptosporidium *positive (Gp III) patients (p > 0.05). However, positive response was observed in significantly higher number of *Cryptosporidium *infected individuals both HIV seropositive (Gp I) and seronegative (Gp III) when compared to *Cryptosporidium *un-infected HIV seropositive (Gp II), patients with other parasitic infections (Gp V) and HIV seronegative healthy (Gp IV) individuals. Our findings are similar to previous study whereby serum IgG positivity was reported to be 100% in AIDS patients with cryptosporidiosis, 87.7% in immunocompetent patients with cryptosporidiosis, 5% in presumably un-infected healthy individuals and 50% in patients with other parasitic individuals [6]. Other studies have also reported positive antibody response in 100% cryptosporidiosis patients with or without HIV [7,8]. Based on assessment of mean OD values, previous studies [24-26] found mean IgG levels higher in sera from HIV infected patients with chronic cryptosporidiosis as compared to healthy controls or HIV infected patients without cryptosporidiosis. In healthy individuals, IgG seropositivity has been found to vary in number of individuals in different parts of the world. IgG antibody response was found positive in 86% in Australia [2], 26% in Britain [3] and 64% in Peru [4] and 64% in Venezuela [4] in healthy individuals. Although, studies regarding IgG antibody response in transplant patients are not available, in the present study, no difference in number of patients with IgG positive response was observed between the two sub-groups in HIV seronegative *Cryptosporidium *positive (post-transplant and presumably immunocompetent) patients (p > 0.05).

In the present study, IgM antibody response was found positive in 2 (18.2%) HIV seropositive *Cryptosporidium *positive (Gp I) and 2 (20%) HIV seronegative *Cryptosporidium *positive (Gp III) including 1 (100%) post-transplant (Gp IIIa) patient while in the *Cryptosporidium *negative groups (Gp II, IV and V), 2 (8%) patients with other parasitic infections showed positive response. No significant difference was observed between number of patients showing positive response in any of the group. Based on assessment of mean OD values, previous studies from France [24-26] found IgM levels higher in sera from HIV infected patients with chronic cryptosporidiosis as compared to healthy controls or HIV infected patients without cryptosporidiosis. Another study found IgM levels higher in sera from children with chronic cryptosporidiosis as compared to healthy controls [27]. IgM serpositivity rate of 15.5% and 19.8% has been reported in healthy children from Peru and Venezuela, respectively [4]. Another study reported serum IgM positivity to be 15.4% in AIDS patients with cryptosporidiosis, 93.3% in immunocompetent patients with cryptosporidiosis, 13% in presumably un-infected healthy individuals and 22.6% in patients with other parasitic individuals [6]. In contrast, some studies have reported IgM positivity rate of 100% in both HIV positive and negative patients with cryptosporidiosis [7,8].

In the present study, IgA antibody response was found positive in 7 (63.6%) HIV seropositive *Cryptosporidium *positive (Gp I) and 5 (50%) HIV seronegative *Cryptosporidium *positive (Gp III) including 1(25%) post-transplant (Gp IIIa) patient while 4 (20%) HIV seropositive *Cryptosporidium *negative, 2 (10%) HIV seronegative *Cryptosporidium *negative healthy and 2 (8%) patients with other parasitic diseases were found positive for IgA response. A positive response was observed in significantly higher number of *Cryptosporidium *infected individuals when compared to *Cryptosporidium *un-infected irrespective of HIV status. In agreement with our findings, a previous study has reported IgA positive response in 100% both in HIV positive and negative patients with cryptosporidiosis [8]. Based on assessment of mean OD values, previous studies found IgA levels higher in sera from HIV infected patients with chronic cryptosporidiosis as compared to healthy controls or HIV infected patients without cryptosporidiosis [24-26] and in sera from children with chronic cryptosporidiosis as compared to healthy controls [27].

The difference in seropositivity in HIV infected as well as healthy subjects in reports from different geographical areas may be attributed to the duration of illness and type of antigen used to study the antibody response.

No reports are available regarding prevalence of anti-*Cryptosporidium *antibodies in India for comparison. However, varying rates of *Cryptosporidium *positivity in both HIV (4.6-12%) and non-HIV (.06-13%) subjects are reported from different geographical locations in India [20].

Sensitivity and specificity of IgG ELISA by using *Cryptosporidium parvum *crude soluble antigen (CCA) was 100% and 95.4%, respectively, in the present study, which is similar to an earlier study [28] which reported the sensitivity and specificity by using two antigens (recombinant form of 27kDa and a partially purified fraction from 17 kDa oocysts antigen) as 90 and 92% and 90 and 94%, respectively. In the present study, sensitivity and specificity of IgM and IgA ELISA was found 19% and 97% and 57.1% and 87.7%, respectively. However, there are no previous reports regarding sensitivity and specificity of IgM or IgA ELISA in cryptosporidiosis.

On comparison of antibody response in HIV seropositive patients with and without diarrhoea, the number of IgG, IgM and IgA seropositive patients with history of diarrhoea was not significantly different from that of patients without history of diarrhoea in both HIV seropositive *Cryptosporidium *positive and HIV seropositive *Cryptosporidium *negative groups. Earlier studies show controversial role of IgG antibodies in cryptosporidiosis. Study done in B-cells depleted BALB/c mouse model indicates that the specific IgG antibody response does not play the major role in the resolution of infection with *Cryptosporidium *[29]. The fact that some AIDS patients with persistent symptomatic infection with *Cryptosporidium *have a high antibody titre to *C. parvum *also supports the theory that specific serum antibody alone is not sufficient to control the infection [9]. In contrast, Experimental study in healthy volunteers reported that seropositivity was higher in persons who were given infection dose >20 fold higher than persons who were seronegative for *Cryptosporidium *[30]. However, the authors suggest that serum antibody may simply be a marker of an effective secretory and/or cellular response to infection. In another study, Frost et al, (2005) reported that in HIV positive individuals, a strong IgG response to the 27-kDa antigen group was associated with a reduced risk of diarrhea [31]. There are no reports available regarding role of IgM antibodies in protection. Previous study shows that HIV positive patients with chronic cryptosporidiosis had higher levels of serum IgA to soluble *Cryptosporidium *antigen compared with HIV positive persons who cleared the infection but secretory IgA antibodies were higher in HIV positive persons who cleared the infection as compared to HIV positive persons with chronic cryptosporidiosis. This suggests that secretory antibody but not serum antibody may be playing role in protection. It has been suggested that secretory IgA is responsible for the recovery from an effective immune response at the mucosal surface [32]. However, the mechanism of diarrhoea in cryptosporidiosis is not well-understood and is suggested to be due to disrupted mucosal architecture and intestinal dysfunction resulting from the infection and the host response to the infection besides other factors [33,34] suggesting that antibody responses may not be the only factors playing significant role in protection from symptomatic cryptosporidiosis, and other innate and cellular immune responses may also be contributing in the protection.

The number of IgG, IgM and IgA positive patients with CD4 counts <200 cells/μl was not significantly different from that of patients with CD4 counts >200 cells/μl in both HIV seropositive *Cryptosporidium *positive and HIV seropositive *Cryptosporidium *negative groups which shows that production of antibodies may not be affected by CD4 counts in HIV patients. In agreement with our findings, a previous study reported no significant difference in peak antibody response to *Cryptosporidium *antigens with respect to HIV status, CD4 cell count or history of diarrhea [35].

## Conclusion

The present study showed specific serum IgG and IgA production in patients infected with *Cryptosporidium*, both HIV seropositive and seronegative as compared to uninfected subjects suggesting induction of *Cryptosporidium *specific humoral immune response in infected subjects. However, there was no difference in number of patients with positive response in HIV seropositive or seronegative groups indicating that HIV status may not be playing significant role in modulation of *Cryptosporidium *specific antibody responses. Sensitivity of IgG ELISA in our study was found to be higher as compared to IgM and IgA ELISA. The number of patients with positive IgG, IgM and IgA response was not significantly different in patients with or without history of diarrhoea in HIV seropositive *Cryptosporidium *positive group thereby indicating that *Cryptosporidium *specific antibody responses may not be necessarily associated with protection from symptomatology.

The limitation of the present study is that we have used crude, soluble antigen of *Cryptosporidium parvum*, which may contain a variety of proteins and nonprotein components, both with and without antigenic properties, and this may be the reason for the differences seen in the findings. Moreover, few conclusions can be drawn because of the small number of subjects in the study with the symptomatic cryptosporidiosis. It will be worthwhile to study and compare the responses of more subjects with the use of more-specific antigenic fractions to ascertain the present findings.

## Competing interests

The authors declare that they have no competing interests.

## Authors' contributions

KK participated in study design, performed analysis and interpretation of data and drafted the manuscript. SK contributed to analysis and interpretation of data and writing of manuscript. AW contributed to analysis and interpretation of data and writing of manuscript. NM conceived the study, participated in its design, contributed to analysis and interpretation of data and corrected draft copies of manuscript.

## Pre-publication history

The pre-publication history for this paper can be accessed here:

http://www.biomedcentral.com/1471-2334/9/179/prepub
